# FPGA-Microprocessor Based Sensor for Faults Detection in Induction Motors Using Time-Frequency and Machine Learning Methods

**DOI:** 10.3390/s24082653

**Published:** 2024-04-22

**Authors:** Roque Alfredo Osornio-Rios, Isaias Cueva-Perez, Alvaro Ivan Alvarado-Hernandez, Larisa Dunai, Israel Zamudio-Ramirez, Jose Alfonso Antonino-Daviu

**Affiliations:** 1 Cuerpo Académico (CA) Mecatrónica, Facultad de Ingeniería, Campus San Juan del Río, Universidad Autónoma de Querétaro, Av. Río Moctezuma 249, San Juan del Río 76807, Querétaro, Mexico; raosornio@hspdigital.org (R.A.O.-R.); isaias.cueva@uaq.mx (I.C.-P.); aalvarado40@alumnos.uaq.mx (A.I.A.-H.); israel.zamudio@uaq.edu.mx (I.Z.-R.); 2 Departamento de Ingeniería Gráfica, Universitat Politecnica de Valencia (UPV), 46022 Valencia, Spain; ladu@upv.es; 3 Instituto Tecnológico de la Energía, Universitat Politecnica de Valencia (UPV), Camino de Vera s/n, 46022 Valencia, Spain

**Keywords:** induction motors, FPGA sensor, machine learning, thermographic images, time domain, time-frequency

## Abstract

Induction motors (IM) play a fundamental role in the industrial sector because they are robust, efficient, and low-cost machines. Changes in the environment, installation errors, or modifications to working conditions can generate faults in induction motors. The trend on IM fault detection is focused on the design techniques and sensors capable of evaluating multiple faults with various signals using non-invasive analysis. The methodology is based on processing electric current signals by applying the short-time Fourier transform (STFT). Additionally, the computation of the mean and standard deviation of infrared thermograms is proposed as main indicators. The proposed system combines both parameters by means of Support Vector Machine and k-nearest-neighbor classifiers. The development of the diagnostic system was done with digital hardware implementations using a Xilinx PYNQ Z2 card that integrates an FPGA with a microprocessor, thus taking advantage of the acquisition and processing of digital signals and images in hardware. The proposed method has proved to be effective for the classification of healthy (HLT), misalignment (MAMT), unbalance (UNB), damaged bearing (BDF), and broken rotor bar (BRB) faults with an accuracy close to 99%.

## 1. Introduction

Induction motors are robust, efficient, low-cost machines that play a fundamental role in industry nowadays. Continuous monitoring of these machines is of great interest due to their widespread use; however, physical changes in the environment, installation errors, or modifications in working conditions can generate faults in them [1]. Induction motor faults can be classified as electrical and mechanical faults, the latter being the most common, representing 55% of faults [2]. Of these, on the one hand, 41% of faults are bearing faults, which can be caused by lack of lubrication, damaged or cracked bearings, rotor overloads, bearing misalignment, vibrations, and motor overheating. On the other hand, 28% are stator faults, 9% are rotor faults, and the remaining 22% take place in other motor components [3]. Continuous monitoring and predictive maintenance are required in the industrial sector in order to ensure availability, reliability, and efficiency. In this regard, diverse non-destructive techniques have been reported in the literature to detect induction motor faults by measuring and processing different physical magnitudes such as mechanical vibration, current, torque, acoustic noise, temperature, speed variation, magnetic flux, and induced voltage [4]. Through the years, researchers have proposed diverse techniques using one, or a combination of two or more, of these physical variables to detect motor faults, as changes or patterns in these variables could point to abnormal operation conditions in the motor, thus aiming to detect these faults.

Techniques for detecting motor faults have been reported in the state-of-the-art literature, classified as time domain, frequency domain, and machine learning techniques. For instance, Nayana and Geethenjali [5] proposed a time domain analysis for detecting faults in inner races, outer races, and rotating components of induction motors. Indicators such as waveform length, slope sign change, simple signal integral, Wilson amplitude, mean absolute value, and zero crossing were calculated from uniform segments of vibration signals. The classification was performed using the Laplacian score (LS) combined with linear discriminant analysis (LDA), obtaining a classification accuracy of 98.94% when analyzing ten bearing fault cases. In another example, Toma and Kim [6] used current signals to calculate ten temporal statistical indicators. The classification was accomplished using Support Vector Machine (SVM), Random Forest (RF), and a KNN (K-nearest-neighbor) algorithms to identify three possible motor states: healthy state, bearing inner race fault, and bearing outer race fault. To give another illustration, Liang et al. [7] proposed a new deep learning-based approach using parallel convolutional neural networks (P-CNN) for bearing fault identification. Two P-CNN branches were built in parallel to extract time domain features of raw vibration signals. These features are fused as inputs in the final classifier. This approach exhibited improved stability and robustness as training dataset size and load conditions varied. Jiang et al. [8] proposed a motor fault diagnostic method based on Feature Incremental Broad Learning (FIBL) and Singular Value Decomposition (SVD). Fault features are extracted from two winding current signals and one acoustic raw signal using particle swarm optimization-variational model decomposition, sample entropy, and time domain statistical features to detect diverse fault states such as short-circuit, mechanical imbalance, bent rotor, bearing raceway defects, and broken bearing balls. A maximum test accuracy of 92.73% was obtained in this work.

Time-frequency domain analysis techniques have also been widely used to detect motor faults in industry. In this regard, Asad et al. [9] performed a broken rotor bar fault modeling and diagnosis by time-frequency analysis of the motor current. A transient current signal was used to perform the analysis using the Discrete Wavelet Transform (DWT). A low-pass Infinite Impulse Response (IIR) filter was used to improve the readability of the time-frequency representations. This research confirmed that this time-frequency approach, combined with machine learning techniques, can detect damaged bars. In another example, Iglesias-Martinez et al. [10] proposed calculating two indicators to discriminate between healthy and faulty rotor conditions in induction motors using stray flux signals. The first one is based on the frequency domain and the bispectrum of the flux signal. A second indicator based on the temporal domain is calculated using the autocovariance function. The results showed that the proposed indicators can provide a criterion to discriminate between healthy and faulty conditions. Similarly, Shao et al. [11] proposed a deep learning approach based on CNNs to detect one healthy condition and five failure conditions. The deep model used multiple sensor signals simultaneously. Explicitly speaking, vibration and current signals were transformed into Time-Frequency Distribution (TFD) images and then used in a CNN to learn discriminative representations from the images. Two different CNN architectures were used, reaching values above 99% of test accuracy in both architectures.

Some authors have also used machine learning techniques using various data types as inputs to detect motor faults. For example, Cao et al. [12] implemented the fault analysis of an induction motor gearbox by studying the time domain vibration signals with a convolutional neural network (CNN) in combination with a transfer learning (TL) technique. The time domain vibration data related to gear fault patterns was converted into graphical images, which serve as input for the CNN. This TL-based approach reduced the training database size, using only ten signals per fault and obtaining a classification accuracy of 99.41%. In another example, Jing et al. [13] used a CNN to fetch indicators from vibration signals to distinguish between seven operation conditions (six faults and one healthy condition) of a planetary gearbox connected to a three-phase induction motor. A comparison between the CNN approach against the Support Vector Machine (SVM) and Random Forest (RF) approaches was performed, concluding that the classification accuracy increased from 92% to 98% using a CNN. Shao et al. implemented a deep belief network (DBN) to diagnose fault conditions in induction motors such as stator short-circuit, unbalance, damaged bearings, broken rotor bar, rotor deflection, and healthy motor using mechanical vibration signals. These signals were transformed using the fast Fourier transform (FFT) to a frequency domain dataset. A 99.9% failure classification accuracy was obtained using this frequency-based approach. In another instance, Toma et al. [14] used genetic algorithms (GA) to analyze the current signals of an induction motor to get the most significant time domain statistical indicators for detecting bearing faults. The selected indicators were used in KNN, decision tree (DT), and RF classifiers, getting a 97%, 98%, and 99% classification accuracy. This approach was applied in three study cases: healthy state, inner race, and outer race fault.

Besides the traditional detection methods based on current or vibration signals, thermography has also been used to detect diverse types of faults on induction motors. For instance, Choudary et al. [15] designed a system for processing infrared thermograms to detect bearing faults. Four failure cases were studied: healthy condition, inner race defects, outer race defects, and lack of lubrication. Fault classification was achieved using an SVM, obtaining an accuracy of 97.9%. In another example, Khanjani y Ezoji [16] used infrared thermograms in a three-phase induction motor to detect electrical faults in the stator windings. Automatic segmentation techniques were used to segment the region of interest (ROI) associated with the motor. A CNN was used to transform the thermograms into representative feature vectors. A KNN and an SVM classifier were used to discriminate among six fault states in the motor. Mahami et al. [17] presented a method using the Speed Up Robust Feature (SURF) approach to process infrared thermograms of an induction motor. This approach was based on calculating invariant descriptors obtained from the thermograms. These descriptors were organized within a Bag of Visual Words (BoVW) to identify eight electrical faults using an extremely randomized tree (ERT). Although high levels of detection accuracy are obtained when reviewing the methods and techniques mentioned above, these works perform classification using an offline approach in order to do so. This implies delayed insights about the motor state and limits or impedes real-time decision-making in case of failure. Consequently, more advanced motor fault detection systems capable of diagnosing different faults commonly appearing in induction motors have emerged in recent years. Furthermore, online detection systems, which are generally implemented using diverse hardware platforms, would also be preferred as they provide several advantages, including early fault detection, remote monitoring, condition-based maintenance, and data analysis to identify trends and patterns in physical variables, potentially saving time and resources in industry environments.

Though most of the works discussed in this section reported results with a good level of classification and diagnosis accuracy in induction motors, the data processing stage was performed in an offline fashion using a PC after the acquisition stage, which, as noted above, implies delayed insights about the motor state and limits and impedes real-time decision-making in case of failure. Consequently, most classification and diagnosis algorithms are implemented in software. Therefore, a hardware-based implementation of such algorithms is desirable because it allows the development of efficient embedded systems for automatic online fault diagnosis in motors. As noted, fusing signal currents and infrared thermograms have not been widely reported for fault diagnosis in motors. In this regard, some researchers have implemented hardware-based online detection systems using field programmable gate array (FPGA) platforms, with advantages such as parallel processing, real-time processing, flexibility, customizability, and high performance. For example, Cureño-Osornio et al. [18] implemented an FPGA-based platform to detect outer race faults in bearings online. The system was implemented using a stray magnetic flux sensor in combination with several IP cores and an embedded processor. The FPGA modules were used to calculate statistical feature values to indicate the bearing condition in the motor, whether a fault is present, and the damage severity in the outer race. Valtierra-Rodriguez et al. [19] presented an FPGA implementation for the complete ensemble empirical mode decomposition (CEEMD) method. This FPGA-based system extracts features to detect broken rotor faults in induction motors. Feature extraction and classification modules were also implemented within the FPGA platform. Results showed a fault detection effectiveness of up to 96%. Karim et al. [20] used an electrical signature analysis based on an FFT algorithm. The stator current signature was used to perform online detection of bearing faults in single-phase induction motors by implementing the frequency domain analysis in an FPGA. Although these developments present an online fault detection approach, it has to be noted that these hardware-based platforms can detect only one type of fault in motors.

This work contributes to developing an FPGA-microprocessor-based sensor platform that runs a series of developed algorithms for the online detection of multiple faults in electric motors. This system uses infrared thermograms and current signals to detect five different failure states, which are healthy (HLT), misalignment (MAMT), unbalance (UNB), damaged bearing (BDF), and broken bar (BRB). The methodology consisted of processing current signals through time domain and time-frequency domain techniques in combination with an analysis of thermographic images that was achieved through diverse image processing techniques. This platform generates a series of indicators from both signals, which are used as inputs of machine learning algorithms to perform fault detection. The construction of this system was achieved through the development of hardware intellectual property cores (IPcores) using a Xilinx PYNQ Z2 board that integrates FPGA along with a microprocessor, thus taking advantage of the acquisition and processing of signals and images in hardware. The IPcores are used to acquire and process the current signals and the thermographic images in order to achieve the automatic diagnosis of the failure state in motors through the implementation and application of supervised classification algorithms.

## 2. Materials and Methods

**Figure 1:**
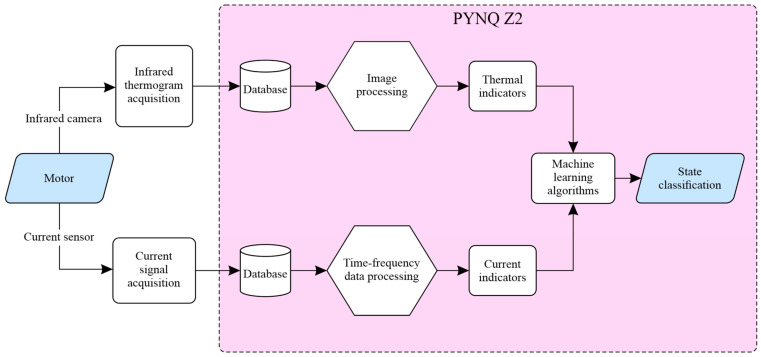
Block diagram of the methodology for detecting faults in induction motors through the FPGA-microprocessor sensor system.

This section describes the methodology employed to develop the sensor system for online fault detection in induction motors. The sensor system was implemented into an FPGA-microprocessor-based platform, making use of indicators obtained from current signals and thermographic images of the motor. Figure 1 presents a block diagram of the methodology of this work. The first stage of the methodology consisted of monitoring temperature and current signals in the induction motor. In this regard, an infrared thermogram acquisition and a current acquisition system were developed in order to monitor both physical variables. Temperature and current data sets were obtained from two different test benches. Acquired data were then stored in two databases using a MicroSD (Micro Secure Digital from Kingston Technology Corporation, Fountain Valley, CA, USA) card in order to be processed in the development board PYNQ Z2 (Xilinx, Inc., San Jose, CA, USA) that integrates the FPGA along with a microprocessor. An FPGA-based platform was selected for the implementation of this work due to its advantages over other kinds of platforms found in the literature that classify faults in motors. First, an FPGA-based hardware platform allows parallel computing, which is highly desirable to reduce computation time. Furthermore, such platforms allow the development and implementation of proprietary hardware modules that can perform specific calculation tasks, which are susceptible to being improved quickly and straightforwardly due to their high reconfigurability. In addition to these factors, the portability of these modules is high, as the description and implementation of the above modules can be performed so that they are not dependent on the FPGA chip vendor. A series of image processing and current signal processing were implemented in this board. Both data processing approaches aim to obtain temperature and current indicators of the motor state that can be analyzed using machine learning algorithms to classify and diagnose the motor state.

### 2.1. Data Processing

Data were acquired from experiments that were performed in two stages. The first stage was exploratory and involved the analysis of thermographic images and current signals from the experiments using different statistical and time-frequency algorithms on a PC using MATLAB (Versión 2020a, The MathWorks, Inc., Natick, MA, USA). The second stage consisted of organizing and implementing these processing algorithms in an embedded hardware-based system using a Xilinx PYNQ Z2 board, which aimed to analyze the thermographic images and the current signals from motors to achieve online fault detection.

**Figure 2:**
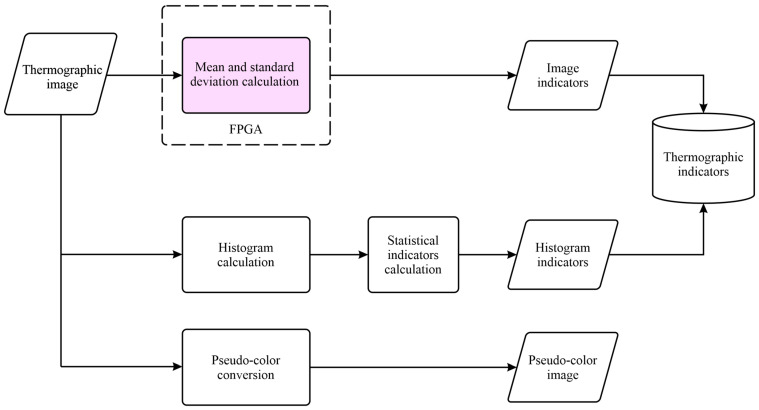
Block diagram of the proposed method for processing thermographic images of the induction motors.

From Figure 1, it is essential to remark that two processing lines were implemented. The first line processed the thermographic images, and the second one processed the current signal from the motor. Figure 2 illustrates the stages of the first processing line. In the first stage, a thermographic image where the temperature was represented using grayscale was captured. After that, the median (μ) and the standard deviation (σ) of the image were calculated within the logic unit of the FPGA from each captured image and used as thermographic indicators, whose equations are shown in (1) and (2), respectively,

(1) $$\mu =\frac{\sum_{y=0}^{h}\sum_{x=0}^{w}I\left(x,y\right)}{w*h}$$

(2) $$\sigma =\sqrt{\frac{\sum_{y=0}^{h}\sum_{x=0}^{w}{\left(\mu -I\left(x,y\right)\right)}^{2}}{w*h}}$$

where $I\left(x,\;y\right)$ is the pixel intensity at location $\left(x,y\right)$, w is the image width, and h is the image height. Simultaneously, a histogram was obtained from each image, which was used to extract statistical indicators from the discrete distribution of intensity levels, as shown in Table 1, where *n* represents the total number of data samples. In this same processing line, the thermographic image was converted to pseudocolor to improve the visual perception of the temperature difference in the image captured.

**Figure 3:**
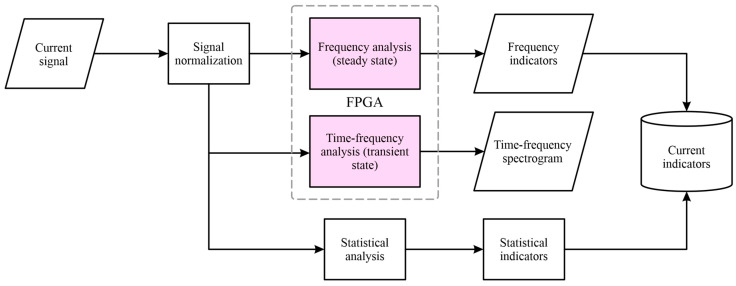
Block diagram of the proposed method for processing the signal current in the induction motor.

Figure 3 shows the stages of the second processing line, which was performed using statistical, frequency, and time-frequency techniques. In the first stage, the current signal was normalized to represent the current in the power line of the motor. After that, a statistical analysis was applied to the normalized current signal using Equations (3)–(17). The frequency analysis was performed using the fast Fourier transform (FFT), and the time-frequency analysis is performed using the short-time Fourier transform (STFT). The FFT and STFT were implemented within the logic unit of the FPGA.

#### 2.1.1. Implementation of Algorithms in FPGA

**Figure 4:**
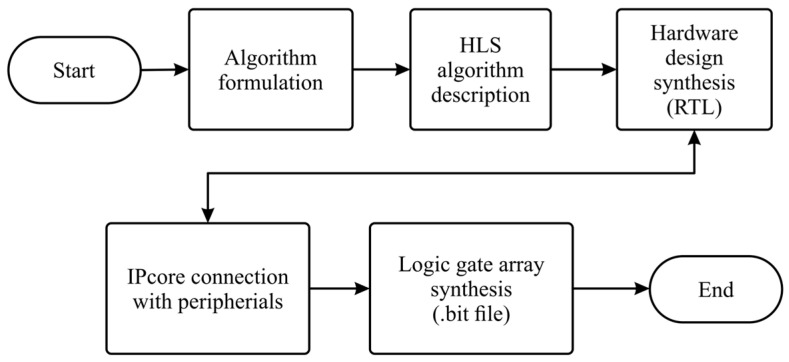
Block diagram of the method for implementing an IPcore in the FPGA of the PYNQ Z2 board.

Figure 4 illustrates the process used to implement IPcores oriented to execute the algorithms and calculations required to be performed within the FPGA. In the first stage, the algorithm was designed. For each IPcore, a hardware description was performed using a High-Level Synthesis (HLS) language. Later, a Register Transfer Level (RTL) abstraction was generated for each description to be implemented on the FPGA. Subsequently, a series of files describing the architecture of each IPcore was imported into Vivado software (version 2022.2, Xilinx, Inc., San Jose, CA, USA) to specify how the described IPcore had to be connected to the board peripherals. Once this connection was set, a bit file was generated to configure the logic gates within the FPGA and implement the IPcores there.

#### 2.1.2. Thermographic-Images Processing IPcore

**Figure 5:**
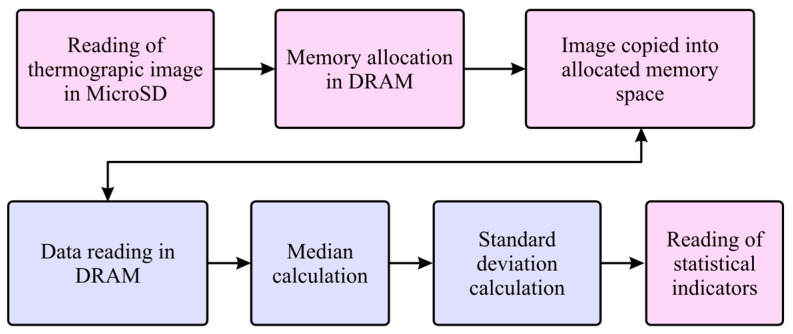
Block diagram with the calculation process to obtain the mean and standard deviation values of a thermographic image in the PYNQ Z2 board.

The first algorithm implemented in the FPGA aimed to calculate statistical indices for the thermographic image. The calculated statistical indicators of the thermographic image were the mean (1) and the standard deviation (2). The algorithm for calculating these indicators on the PYNQ Z2 board was implemented following the diagram shown in Figure 5, where the blocks in pink were executed on the board microprocessor, and the blocks in purple were executed on the FPGA. In the first step, thermographic images of the induction motor acquired during the experiments were saved to a MicroSD memory that had to be placed on the PYNQ Z2 card. The intensity values of the thermographic images were reassigned to be in a 0–255 range for storing the information in 8-bit data packets and saved in a jpeg format. After this, a space was allocated in a DRAM memory integrated within the board to store the image data, and a copy of the image data was made in the allocated space. The DRAM memory served as the communication interface between the microprocessor and the FPGA of the development board. 

**Figure 6:**
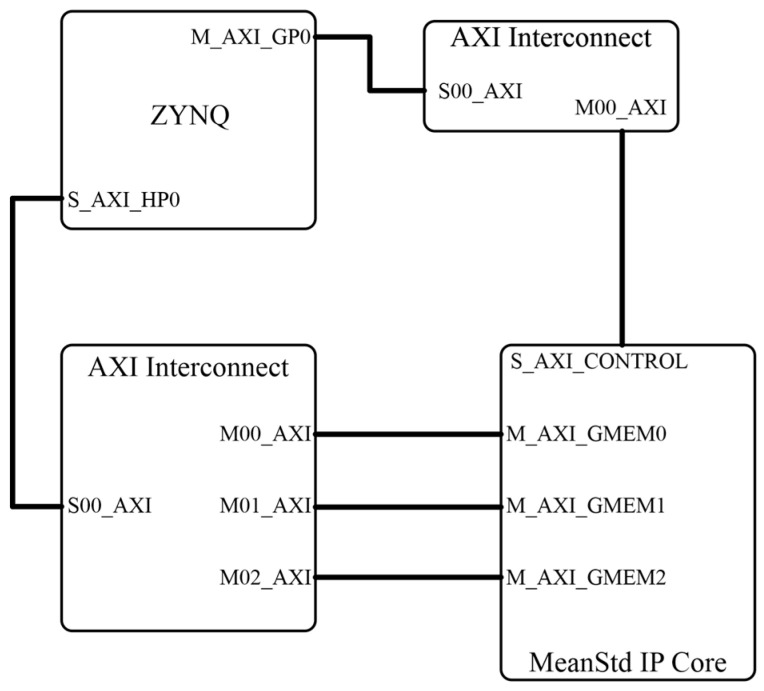
Block diagram showing the connections between the hardware components of the statistical calculation algorithm.

The peripheral block diagram for implementing this IPcore and its interconnection with the ZYNQ main chip is shown in Figure 6. This block diagram shows the interconnection topology used to link the MeanStd IPcore located in the lower right corner of the diagram with the ZYNQ chip located at the top left of the diagram. The AXI protocol was used in order to implement this interconnection. This protocol defines the IP block interface using a master–slave scheme. Two interconnected blocks (AXI Interconnect blocks at the top right and bottom left) were required to link the MeanStd IPcore with the ZYNQ chip which is within the PYNQ Z2 board. AXI ports were defined through three master interfaces, represented by the three lines connecting the MeanStd IPcore with the AXI Interconnect block at the bottom left of Figure 6. One of these interfaces was dedicated to reading the image data from the card DRAM memory. The other two interfaces were dedicated to storing the calculation results from the statistical indicators.

#### 2.1.3. Current Signal Processing IPcore

**Figure 7:**
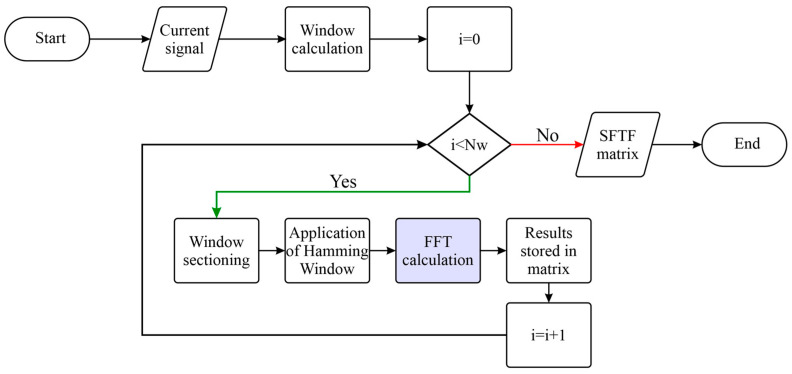
STFT calculation method with the FPGA unit of the PYNQ Z2 board.

The algorithm implemented in the FPGA for current signal processing was the fast Fourier transform (FFT). Two FFT IPcores were designed; one focused on the steady-state frequency analysis of the current signal, and the other focused on the time-frequency analysis of the current signal. In the case of the frequency analysis, an IPcore was designed to calculate the FFT with a fixed size of $N_{FFT}=2^{16}=65536$. The other IPcore focused on the STFT calculation. For the design of this IPcore, a fixed size was determined $N_{FFT}=2^{10}=1024$. Figure 7 illustrates the STFT calculation of a current signal with the FFT IPcore in the FPGA logic unit of the PYNQ Z2 board. The first step consisted of reading the current signal from the MicroSD memory. Then, the signal was normalized in a Python-based framework that operated within the microprocessor to represent the real magnitude of the measured current. After the signal was read, the signal had to be divided into a certain number of windows, which were calculated using Equation (18) in order to perform a short-time analysis

(18) $$NW=\lceil \frac{k-N_{FFT}}{\left(1-Ov\right)N_{FFT}}\rceil$$

where k is the initial length of the signal, $N_{FFT}$ is the FFT size, and Ov is the superposition value of the window per unit. After determining the number of windows into which the signal was to be divided, the clipped window of the signal was multiplied with a Hamming window of size equal to $N_{FFT}$. Later, the normalized and preprocessed current signal was copied to an allocated space in the DRAM memory of the board, from where the Direct Memory Access (DMA) module of the IPcore could access the signal values. The IPcore calculated the FFT of each of the NW windows of the current signal. Finally, a matrix containing the FFT of each analyzed current signal window was generated.

### 2.2. Experimental Setup

#### 2.2.1. Test Bench with WEG Motor

**Figure 8:**
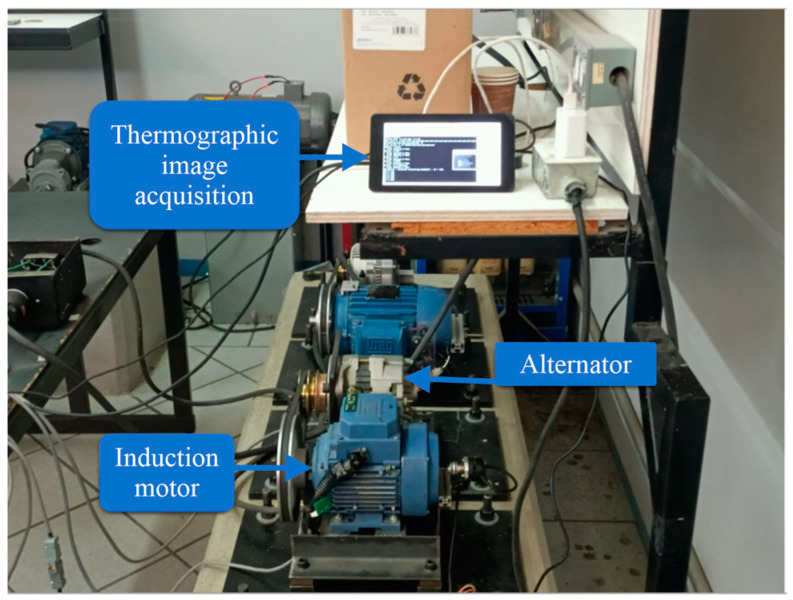
Test bench with a WEG three-phase induction motor.

**Figure 9:**
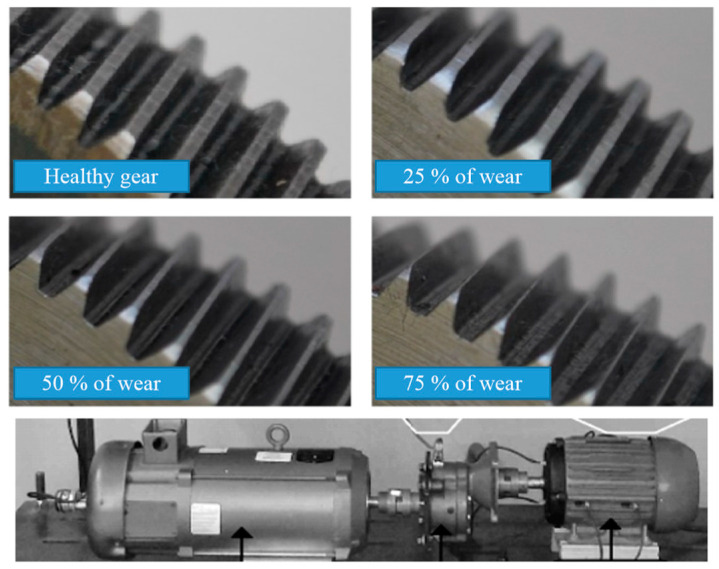
Fault caused by gearbox wear connected to a WEG motor.

The first test bench used in the experiments shown in Figure 8 was located in the laboratory of electrical machinery of the Faculty of Engineering at Universidad Autónoma de Querétaro, San Juan del Río Campus. A WEG three-phase induction motor model 3F A.E. 00136AP3E48TCT (Jaragua do Sul, Brazil) was used in this test bench; specifications of this motor are shown in Table 2. The motor was connected to an alternator that served as a mechanical load. The connection between the motor and the alternator was made by a set of pulleys and a plastic power transmission belt. The experiments on this test bed were performed with a direct start at an operating frequency of 60 Hz. To measure the stable temperature of the motor, each test had a duration of 90 min. The thermographic image acquisition system shown in Figure 9 captured an image of the motor every 10 s, thus obtaining a database of 540 thermographic captures per study case. Additionally, the acquisition of current signals from the induction motor was performed. The sampling frequency of the current signal acquisition was 4 kHz. For each study case, five trials were conducted, each one with a duration of 30 s. The first 10 s of the signal corresponded to the motor start-up stage, and the remaining 20 s corresponded to the steady state of the current signal. Five cases of failure in the induction motor or the components connected to it were studied in this test bench: healthy, bearing defect, broken bars, unbalance, and misalignment. The study cases are described in Table 3; each has a label associated with it for an abbreviated reference to each failure case.

Different severities were considered for two of the case studies shown in Table 3, namely, bearing failure and broken bar failure. Tests were performed for the bearing failure case with five severities of damage in the outer race of metallic bearings. The severities were induced by drilling on the outer race with the following diameters: 1 mm, 2 mm, 3 mm, 4 mm, and 5 mm. Five SKF model 6203 metallic bearings were used. In the case of the broken bar study, three severities were considered during the experiments: half-broken bar, one broken bar, and two broken bars. For the gearbox failure (GRF) case, a gearbox was connected to the WEG induction motor instead of the belt pulley connection. Four gearbox wear severities were considered: healthy, 25% wear, 50% wear, and 75% wear. Figure 9 shows the induced wear on the gears and the configuration of the test rig.

#### 2.2.2. Induction Motor 2 Testbench

**Figure 10:**
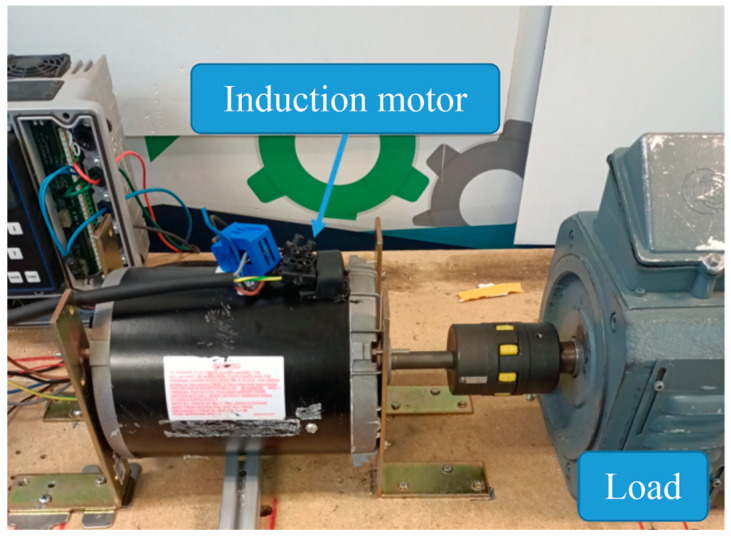
Test bench with IM2 connected to a DC motor (load).

The second test bench used for experiments in this work was A 1 HP three-phase induction motor, whose specifications are shown in Table 4. The induction motor 2 (IM2) was connected to a DC motor, as shown in Figure 10. A rheostat connected to the DC motor stator was used to control the load level of the induction motor. This rheostat made it possible to perform experiments under four different load levels: nominal load (100%), load at 75% of nominal load, load at 50% of nominal load, and no load (0%). The tests were performed in direct-start at an operating frequency of 50 Hz and a supply voltage of 220 V. Six cases of induction motor mounting failure were studied in this test bench: healthy, unbalance (with two severities), horizontal misalignment, vertical misalignment, and bad mounting due to a loose screw. Table 5 describes the case studies and lists the labels to refer to them. Since the IM used in these tests did not have a cooling system, the duration of the tests for the acquisition of thermographic images was limited to 5.5 min, with an idle time of 3 min between tests. The sampling rate of the thermographic camera in these experiments was nine frames per second (fps). For the acquisition of the electric current signals, 30 s trials were performed; the first 10 s corresponded to the motor start-up, and the remaining 20 s corresponded to the steady state of the current. The signals were acquired with a sampling frequency of 10 kHz.

### 2.3. Data-Acquisition Systems

#### 2.3.1. Acquisition of Thermographic Images

**Figure 11:**
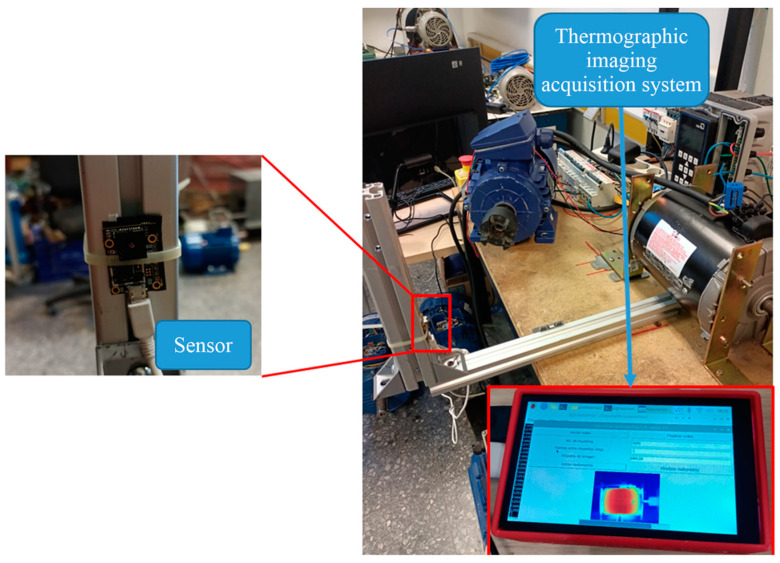
Acquisition system of infrared thermographic images installed on the test bench.

The acquisition of thermographic images was achieved with a system that integrated a FLIR Lepton 3.5 sensor (Teledyne FLIR LLC, Wilsonville, OR, USA) with a Raspberry Pi 4 computer board (Raspberry Pi Foundation, Cambridge, England, UK). This system allowed the acquisition and storage of infrared thermograms with a variable sampling rate. The limiting sampling rate was nine fps, given the technical limitations of the infrared sensor. Figure 11 shows the infrared thermogram acquisition system on the test bench. On the other hand, Table 6 shows the technical specifications of the FLIR Lepton 3.5 infrared sensor. The thermographic image stored the temperature values of the scene according to Equation (19)

(19) $$V\left(x,y\right)=100*T_{K}\left(x,y\right)$$

#### 2.3.2. Current Signal Acquisition

**Figure 12:**
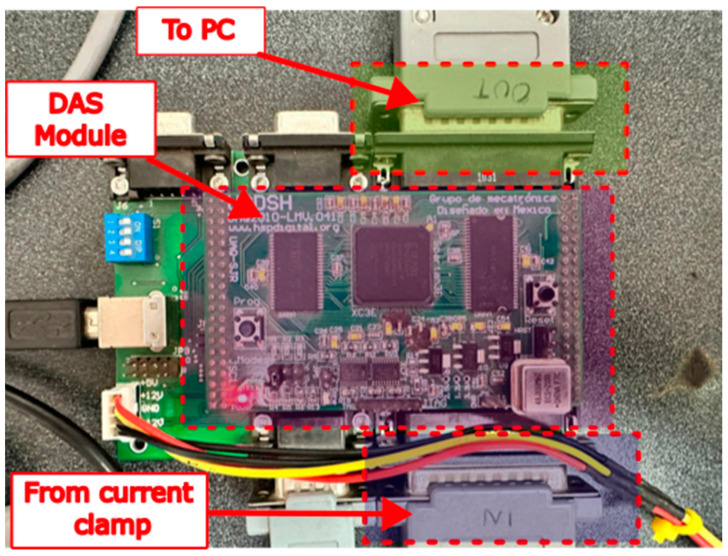
Data acquisition system used to measure signal currents in the WEG motor.

**Figure 13:**
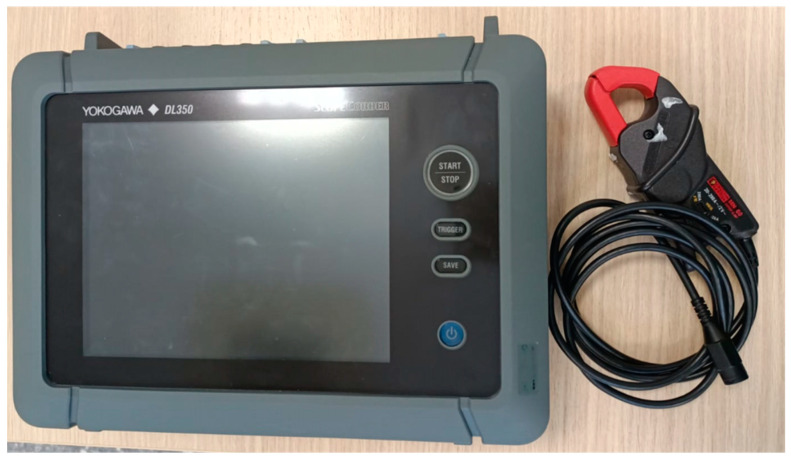
Data acquisition system used to measure current signals in the IM2.

In the experiments carried out in the first test bench with the WEG motor, the motor current signals were acquired using the data acquisition system (DAS) shown in Figure 12. This DAS (Universidad Autonoma de Queretaro, Queretaro, Mexico) was composed of an FPGA controlling an ADC ADS7841 from Texas Instruments (Dallas, TX, USA), one connector to input the current clamp signals, and one connector which enabled the sending of the captured data to a PC for further analysis. The ADC had a 12-bit resolution. Each sensor received the signal from one of the three-phase motor supply lines; the fourth sensor measured the current signal on the grounding line. The sampling frequency of the DAS was set at $F_{s}$ = 4000 Hz. In the experiments with the second test bench based on the IM2, the current signal of only one of the three-phase power supply lines was acquired for the induction motor. The data acquisition was performed with a Yokogawa digital oscilloscope (Tokyo, Japan) and a current clamp, shown in Figure 13. The current clamp had a 10 kHz bandwidth, and could be measured in a range from 0.5 A to 200 A. The sampling frequency for the current signal of the IM2 was set at $F_{s}$ = 10,000 Hz.

## 3. Results and Discussion

This section aims to address the results obtained in the implementation of the algorithms in the FPGA logic unit of the PYNQ Z2 development board, a discussion of the acquired data, the results obtained by processing the acquired data, and the results obtained in the application of machine learning algorithms for data classification.

### 3.1. Algorithm Implementation on the FPGA

Table 7 shows the resource utilization of the PYNQ Z2 FPGA unit required by the algorithm for calculating statistical indicators (mean and standard deviation). The hardware description for these statistical indicators reduced the computational time needed to complete the assigned tasks. This time reduction was observed by comparing the performance when running the algorithm on the PYNQ Z2 microprocessor and the performance when running the algorithm on the FPGA unit in conjunction with the PYNQ Z2 microprocessor. The indicator calculation algorithm was tested with a database of 900 thermographic images, which were processed in 1.4 s by the microprocessor and 346 ms by the IPcore implemented in the FPGA unit. On the other hand, Table 8 shows the resource utilization in the FPGA logic unit required by the IPcore for the FFT calculation of a discrete signal. Significantly, implementing the IPcore in the FPGA reduced the computation time compared with the ARM microprocessor integrated into the development board. Using the NumPy Python library implementation, the time taken by the microprocessor to calculate the FFT was 385 μs. On the other hand, the IPcore implementation on the FPGA calculated the FFT in 79.6 μs.

### 3.2. Data Acquisition

**Figure 14:**
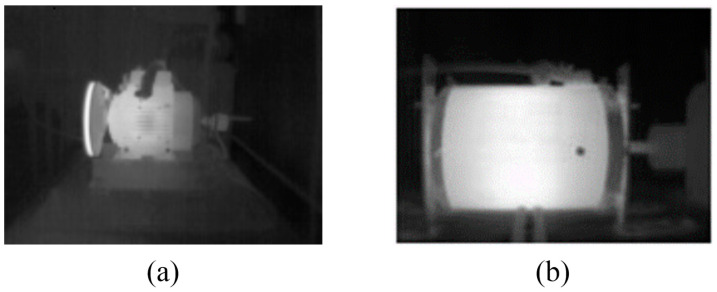
Thermographic images captured of induction motors: (**a**) WEG motor, (**b**) IM2.

The acquisition of the infrared thermograms of the motor and the preprocessing to visualize the information in the form of a grayscale image was performed satisfactorily, as shown in Figure 14. These images are thermographic representations of the studied motors, associating different levels of gray to temperature values. On the one hand, hotter regions are represented with tones closer to white; and on the other hand, colder regions are represented with gray tones closer to black. Therefore, white tones represent the highest temperature within the image, and black tones represent the lowest temperature. Figure 14a shows the thermographic representation of the WEG motor side during the healthy machine case study tests. In turn, Figure 14b shows the thermographic representation of the IM2 side during the healthy condition tests. 

**Figure 15:**
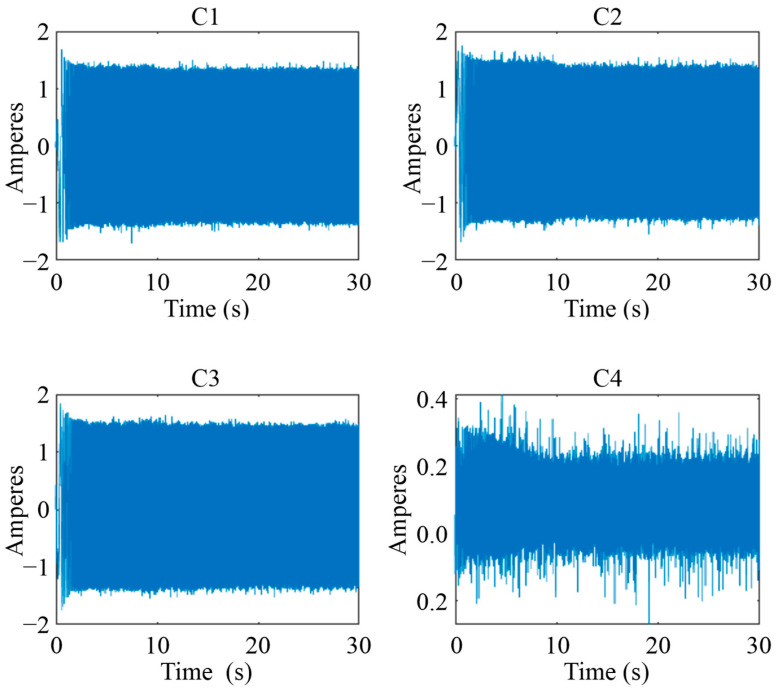
Current signals acquired from the WEG motor in healthy state.

Four current signals were acquired from the WEG motor during the tests with a sampling frequency $F_{s}$ = 4000 Hz. Figure 15 shows the time evolution of the four acquired signals. Signals C1, C2, and C3 correspond to the current measured on the three-phase motor supply lines. Signal C4 corresponds to the current measured in the grounding line.

### 3.3. Data Processing

#### 3.3.1. Analysis of Thermographic Images

The thermographic images of the induction motors were analyzed by calculating two statistical indicators of the image: the mean and the standard deviation. The histogram of the thermographic images was also obtained, and the statistical indicators from Equations (3)–(17) were calculated. Additionally, a pseudocolor palette was applied to the thermographic images.

##### WEG Motor

**Figure 16:**
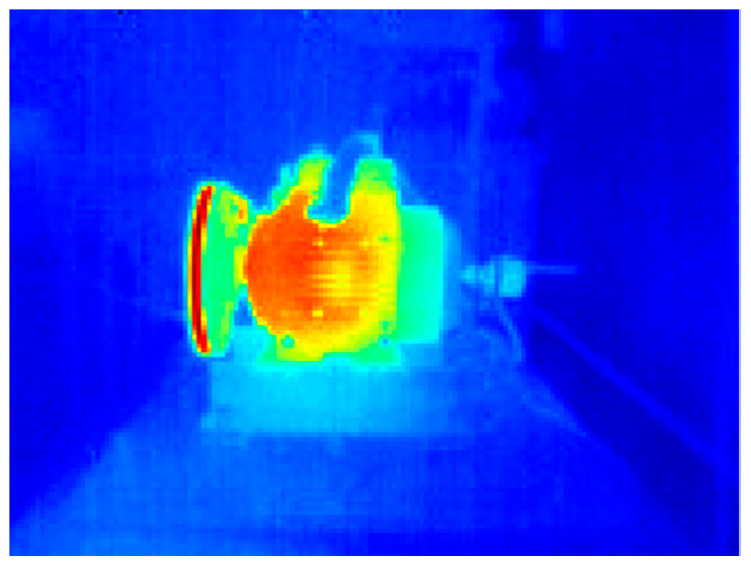
Thermographic image of the WEG motor with applied pseudocolor palette.

**Figure 17:**
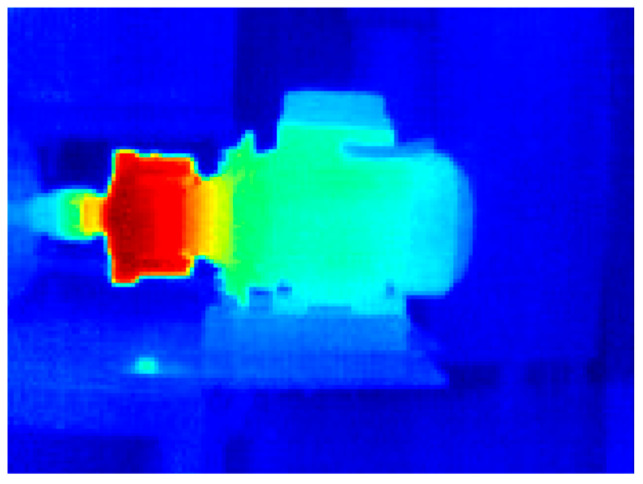
Thermographic image of the WEG motor with gearbox after applying the pseudocolor palette.

Figure 16 shows the application of the pseudocolor palette to the infrared thermographic image of the WEG induction motor in a healthy condition; thanks to the pseudocolor palette, the highest temperature region in the motor can be qualitatively appreciated. Figure 17 shows the pseudocolor application to capture the WEG motor connected to the gearbox in a healthy condition. In these graphic representations shown in Figure 16 and Figure 17, which aim to improve the visualization of results obtained from thermographic images, a pseudocolor palette was applied to the image to represent the highest temperatures in reddish tones. In contrast, the lowest temperatures are graphically represented in blueish tones. For instance, the gearbox of the WEG motor shown in Figure 17 presents the highest temperatures in this thermographic image with applied pseudocolor. 

**Figure 18:**
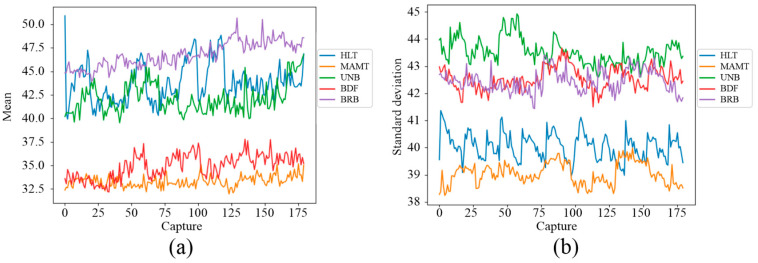
Evolution of thermal indicators in the WEG motor: (**a**) mean, (**b**) standard deviation.

Likewise, the IPcore was used to calculate statistical indicators for the quantitative analysis of the thermographic captures. A database of quantitative indicators of the images was obtained by calculating the mean and standard deviation of the thermographic images. These indicators were plotted to observe their behavior in five tests with different failure states. Figure 18a shows the evolution in the steady state of the mean in the thermographic image and Figure 18b shows the steady state evolution of the standard deviation in the thermographic image. Each failure is associated with a different color within these figures. For instance, Figure 18a shows that the BDF and BRB indicators of the mean are almost overlapped in this plot. Furthermore, as observed in this plot, it seems that two groups of failures can be inferred from these results. The first consisted of the BDF and MAMT failures, and the second one was the HLT, UNB and, BRB failures that show overlap within some periods of time. On the other hand, two groups of failures can be observed from Figure 18b; the first consists of indicators corresponding to HLT and MAMT failures, which are in the lowest part of the plot, presenting little overlap. The second group consisted of the standard deviation indicators for the UNB, BDF, and BRB failures at the top of the plot. It can also be observed that the evolution of BDF and BRB indicators overlap almost all the time (*x*-axis). 

**Figure 19:**
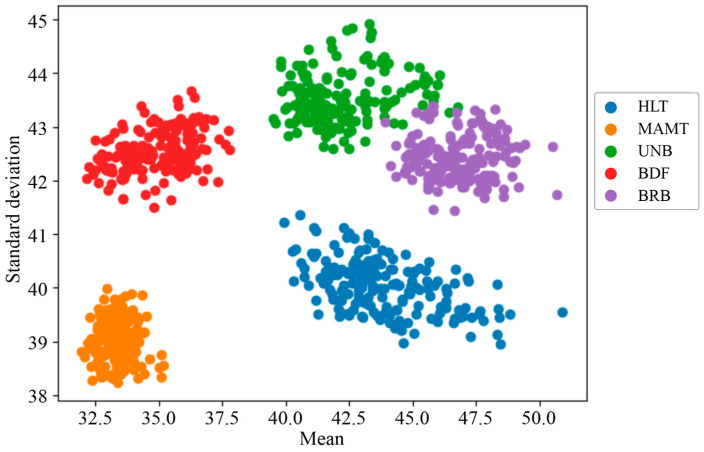
Data groups of statistical indicators extracted from the thermographic images in the WEG motor.

Additionally, the two variables (median and standard deviation) were plotted with respect to each other from which it was observed that the indicators were grouped according to the failure state to which they belonged (Figure 19). The failure states analyzed were healthy (HLT), misalignment (MAMT), unbalance (UNB), damaged bearing (BDF), and a broken bar (BRB). This plot clearly shows five differentiable groups of statistical indicators plotted using different colors, each associated with a failure. However, although clear separation is noticeable, little overlap is present between the UNB and the BRB failures. Additionally, this plot shows the relevance of using two variables within the scope of this work, as the evolution of single statistical indicators such as mean and standard deviation was insufficient to distinguish each failure case clearly.

**Figure 20:**
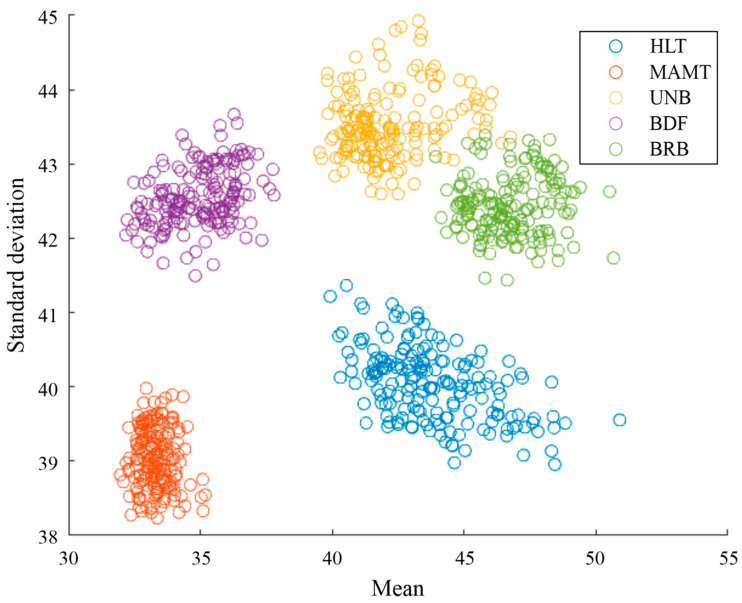
Results from mean and standard deviation analysis in MATLAB.

The data processing of the thermographic images of the WEG motor in the FPGA unit of the Xilinx PYNQ Z2 board was validated by comparing the results obtained with the analysis of the mean and standard deviation of the thermographic images in MATLAB. Figure 20 shows the statistical indicators grouped according to the motor failure case. It can be observed that the results in Figure 19 and Figure 20 are the same, which indicates that the analysis performed on the PYNQ Z2 board was correct.

##### IM2

**Figure 21:**
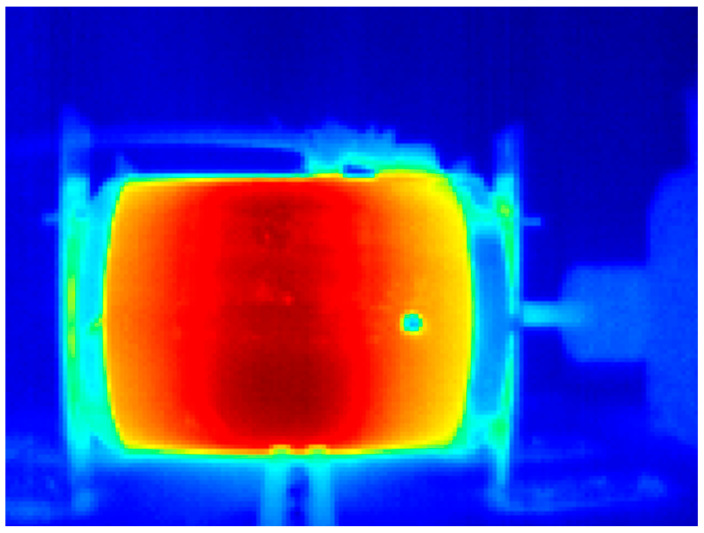
Infrared thermogram of the IM2 with applied pseudocolor palette.

**Figure 22:**
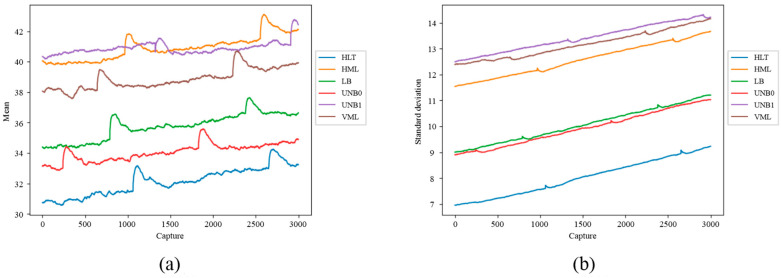
Evolution of thermographic indicators in IM2: (**a**) mean, (**b**) standard deviation.

**Figure 23:**
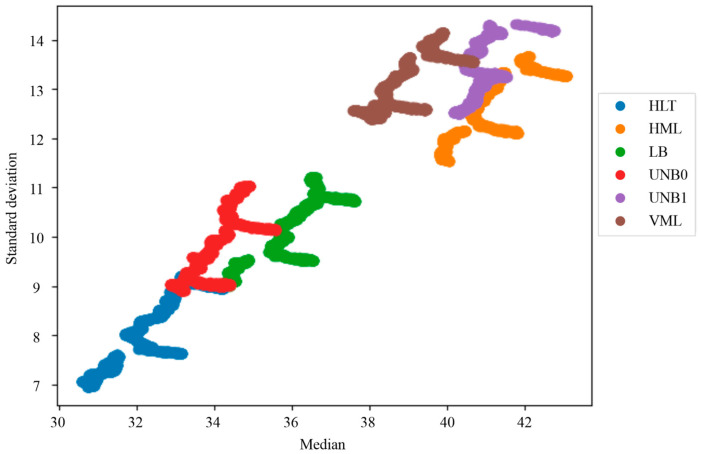
Groups of statistical indicators extracted from the thermographic images in the IM2.

Figure 21 shows the application of the pseudocolor palette to the infrared thermographic image of the IM2 in a healthy condition; thanks to the pseudocolor palette, the highest temperature region in the motor can be qualitatively appreciated in reddish tones. In this case, a vertical reddish band in the central region of the motor indicates the region with the highest temperatures. The IPcore implemented in the FPGA was used to calculate the two statistics of the set of thermographic images acquired, thus obtaining a database of quantitative indicators of the images. Figure 22a shows the evolution of the mean of the thermographic images, while Figure 22b shows the evolution of the standard deviation of the thermographic images. In the plot of Figure 22a corresponding to the mean indicator, some interesting aspects can be appreciated. In the first place, a better distinction can be made between the different failures in the case of motor IM2 in comparison with the equivalent result of the WEG motor using the evolution of this statistical indicator over time, as only two failures out of six overlap here. However, that is not the case for the evolution in the standard deviation over time shown in Figure 22b, as more overlap is present. In fact, three groups of failures can be observed in the plot, with very high overlap in the case of UNB0 and LB. Finally, Figure 23 shows the data grouping obtained using the two statistical indicators as a reference. The case studies shown are healthy motor (HLT), horizontal misalignment (HML), loose bolt (LB), minor unbalance (UNB0), major unbalance (UNB1), and vertical misalignment (VML). As observed in this plot, two groups of three failures are formed. The first group, located in the lower-left part of the plot, corresponds to the HLT, UNB0, and LB failure indicators, and the second group is located in the top-right part of the plot, containing the VML, UNB1, and HML failure indicators. It is interesting to pinpoint that the second failure group corresponds to the mechanical connection of the motor with the load.

#### 3.3.2. Current Analysis

The analysis of the current signals of the motors was performed with three approaches: first, a temporal statistical analysis of the acquired signals was performed with the indicators of Equations (3)–(17); second, the frequency analysis of the signals was performed with the FFT; and finally, time-frequency spectrograms were obtained with the STFT technique.

##### WEG Motor

**Figure 24:**
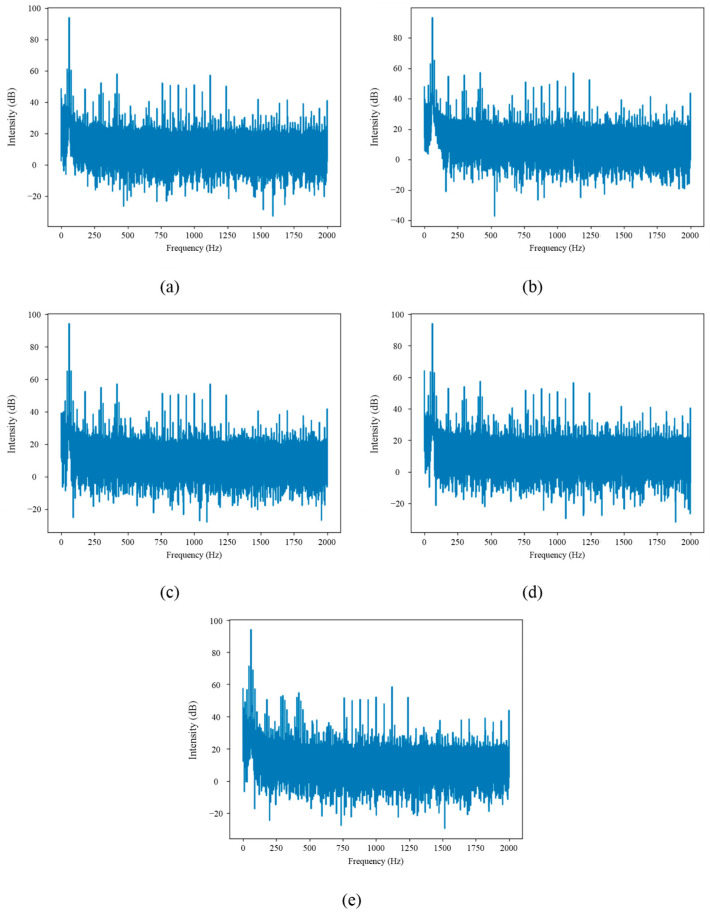
Frequency analysis of the current signal in the WEG motor obtained with the FFT for: (**a**) healthy motor, (**b**) half-broken bar, (**c**) fully broken bar, (**d**) unbalance, and (**e**) misalignment.

**Figure 25:**
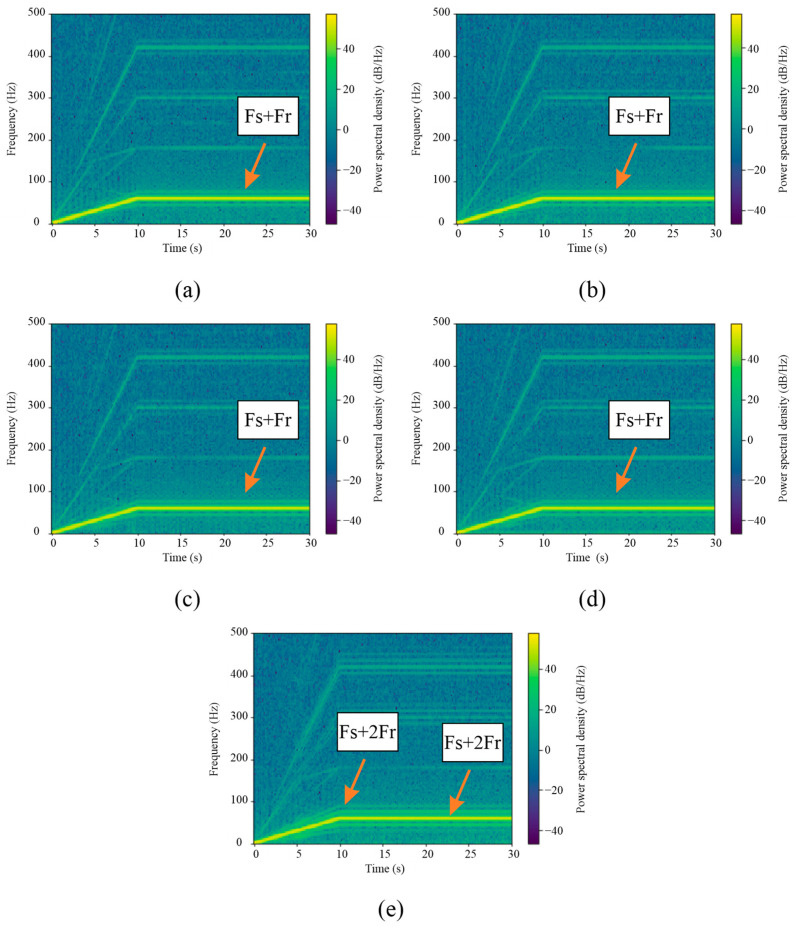
Current-signal spectrograms from the WEG motor: (**a**) healthy motor, (**b**) half broken bar, (**c**) fully broken bar, (**d**) unbalance, and (**e**) misalignment.

In the frequency analysis, the fast Fourier transform (FFT) of the motor current signals was calculated. Figure 24 shows the graphical representation of the results obtained after calculating the FFT of the signals for different fault cases. The time-frequency analysis performed in the transient (10 s duration) and stationary (20 s duration) regime in the current signals of the WEG motor is observed in the spectrograms shown in Figure 25. The spectrograms shown in Figure 25 were calculated by taking a sampling frequency $F_{s}$ = 4000 Hz, a window size of $N_{FFT}$ = 1024 samples, and a window overlap of 75%. These spectrograms were generated on the PYNQ Z2 development board with the IPcore implemented for FFT calculation.

### 3.4. Failure Status Classification

Different classification models were trained with machine learning algorithms in MATLAB software, and the mean and standard deviation data of the thermographic images were used as indicators of the machine failure state. For training the algorithm, a total of 900 thermograms are extracted from the measurement in the experimental tests (180 (samples) by 5 (machine states)). From the 180 thermograms obtained for each machine state (classification goal), 126 are used for the training of the classification algorithm and 54 for validation. One of the trained models is a Weighted K-Nearest Neighbor (KNN) classifier with k=10, considering the Euclidean distance between points, and using the inverse of the square of the distance for weighing. The training result gave a 99.4% accuracy in predicting the failure cases. The training was validated by performing a 5-iteration cross-validation, obtaining the confusion matrix in Table 9. Another machine learning classifier model trained with this data set was a Linear Support Vector Machine model. The classifier model obtained from the training gave an accuracy of 99.0% in predicting the five failure cases. Table 10 contains the confusion matrix of the SVM classifier model. 

As illustrated in Figure 24 and Figure 25, the FFT and STFT computed using the developed IPcore demonstrates robust and reliable results. This figure specifically highlights how, in instances of mechanical misalignment, there is a noticeable amplification of fault-related frequency components. These components correspond to the frequency $F_{MAMT}=Fs\;\pm k\cdot Fr$, where Fs represents the power supply frequency, *Fr* is the rotor rotational frequency, and k is an integer. This amplification is critical for the early detection of mechanical faults, ensuring timely maintenance and system integrity. The clarity and precision of the spectral lines within the STFT visualization confirm the effectiveness of the IPcore in accurately identifying frequency variations that signal potential mechanical issues.

Additionally, Table 11 compares the most recent studies reported in the literature concerning the detection of electromechanical faults in induction motors. This comparison encompasses each methodology’s main techniques, the analyzed physical quantities, and the accuracy rates achieved. As can be observed in the table, many of the reported studies rely on a single quantity for classification purposes, which tends to limit the range of faults that can be classified. It is noteworthy, however, that as the number of physical quantities analyzed increases—whether it be stray flux, current, vibrations, or acoustic signals—the overall efficiency of the method tends to improve, and the variety of faults that can be accurately classified also expands. A significant insight revealed by the table is that the proposed methodology can classify a wide array of faults using just a single, non-invasive physical quantity, namely infrared thermography. Furthermore, most reviewed methodologies employ complex algorithms that typically require substantial computational resources regarding memory and processing power to be implemented on an electronic device capable of generating continuous automatic diagnostics. Herein lies a significant opportunity for the proposed methodology, given that the methods it uses are straightforward to implement and require low computational resources; therefore, this makes it an excellent alternative for implementation on programmable logic devices such as FPGAs, as this paper has shown. This characteristic enables the development of devices capable of continuously generating timely, non-invasive diagnostics.

## 4. Conclusions

This work reports the development of an FPGA-microprocessor-based sensor to detect faults in induction motors. This development was achieved by implementing statistical and time-frequency analysis techniques as hardware IPcores that work in the logic unit of an FPGA. These specific-purpose IPcores were used together with some algorithms implemented within an ARM microprocessor within the same embedded system. The IPcore version of the algorithms was executed significantly faster than the same algorithms executed in the microprocessor of the embedded system. Furthermore, the usage of computational resources within the FPGA was low (15% per IPcore). The proposed methodology for processing thermographic images acquired with a low-cost infrared sensor is effective for acquiring significant statistical indicators for identifying different fault cases, with an accuracy close to 99% with machine learning classifiers such as KNN and SVM. In this regard, the proposed algorithms and statistical indicators of this work allowed for an improvement in the distinction and correct classification of the different failures in induction motors when combining multiple statistical indicators instead of a single one. Additionally, the analysis of both thermographic images and current signals provides valuable insights into the condition of the motors under study. By examining the statistical indicators derived from thermographic images, such as the mean and standard deviation, and utilizing pseudocolor palettes for enhanced visualization, distinct patterns emerge corresponding to various failure states. For instance, in the case of the WEG motor, the evolution of these indicators over time reveals discernible groupings associated with specific faults, demonstrating the effectiveness of this approach in fault diagnosis. On the other hand, the frequency analysis of current signals through techniques like the fast Fourier transform (FFT) and the short-time Fourier transform (STFT) provides additional diagnostic capabilities. Notably, the STFT, computed using the developed IPcore, exhibits robustness in identifying fault-related frequency components, particularly in cases of mechanical misalignment.

Additionally, motor current signals were processed by time-frequency techniques that generated bidimensional spectrograms that show the evolution of the signal frequency components with time. The implemented algorithms were used to perform experiments in two different test benches, processing both infrared thermographic images and current signals from both motors. However, it is relevant to point out that, given the differences in the acquired signals, the implementation of such algorithms in the FPGA is robust enough to deliver satisfactory and accurate results compared to software-based implementations such as MATLAB. As this system was implemented in an FPGA-based platform, future work might reconfigure the system to consider more fault cases in induction motors or to implement more machine learning techniques that could use the time-frequency spectrograms of current signals, such as CNNs.

## Figures and Tables

**Table 1:** Equations for calculating the statistical indicators.

Statistical Indicator	Equation	
Mean	$\bar{x}=\frac{\sum_{1}^{N}x\left(n\right)}{N}$	(3)
Maximum value	$\hat{x}=\max\left(x\right)$	(4)
Root mean square (RMS)	$RMS=\sqrt{\frac{1}{n}\cdot \overset{n}{\underset{k=1}{\sum }}{\left(x_{k}\right)}^{2}}$	(5)
SMR	$SMR={\left(\frac{1}{n}\cdot \overset{n}{\underset{k=1}{\sum }}\sqrt{\left|x_{k}\right|}\right)}^{2}$	(6)
Standard deviation	$\sigma =\sqrt{\frac{1}{n}\cdot \overset{n}{\underset{k=1}{\sum }}\sqrt{{\left(x_{k}-\bar{x}\right)}^{2}}}$	(7)
Variance	$\sigma^{2}=\frac{1}{n}\cdot \overset{n}{\underset{k=1}{\sum }}\sqrt{{\left(x_{k}-\bar{x}\right)}^{2}}$	(8)
RMS shape factor	$SF_{RMS}=\frac{RMS}{\frac{1}{n}\cdot \sum_{k=1}^{n}\left|x_{k}\right|}$	(9)
SMR shape factor	$SF_{SRM}=\frac{SRM}{\frac{1}{n}\cdot \sum_{k=1}^{n}\left|x_{k}\right|}$	(10)
Crest factor	$CF=\frac{\bar{x}}{RMS}$	(11)
Latitude factor	$LF=\frac{\hat{x}}{SRM}$	(12)
Impulse factor	$IF=\frac{\hat{x}}{\frac{1}{n}\cdot \sum_{k=1}^{n}\left|x_{k}\right|}$	(13)
Skewness	$S_{k}=\frac{\sum_{k=1}^{n}{\left(x_{k}-\bar{x}\right)}^{3}}{\sigma^{3}}$	(14)
Kurtosis	$K=\frac{\sum_{k=1}^{n}{\left(x_{k}-\bar{x}\right)}^{4}}{\sigma^{4}}$	(15)
Fifth moment	$5thM=\frac{\sum_{k=1}^{n}{\left(x_{k}-\bar{x}\right)}^{5}}{\sigma^{5}}$	(16)
Sixth moment	$6thM=\frac{\sum_{k=1}^{n}{\left(x_{k}-\bar{x}\right)}^{6}}{\sigma^{6}}$	(17)

**Table 2:** WEG motor specifications.

Parameter	Value	Units
Supply voltage	208–230/460	V
Nominal speed (@60 Hz)	3355	rpm
Power	746	W
Nominal efficiency	75.5	%
Nominal current (@460 V/60 Hz)	1.4	A
Power factor	0.87	
Weight	9	kg

**Table 3:** Study cases for WEG induction motor.

Study Case	Label	Description
Healthy	HLT	Healthy motor and kinematic chain
Bearing defect	BDF	Motor bearing defect
Broken bars	BRB	Broken bars in the motor rotor
Unbalance	UNB	Mechanical unbalance in the motor axis
Misalignment	MAMT	Misalignment between the motor and the load
Gearbox wear	GRF	Wear in the gearbox connected to the motor

**Table 4:** Specifications of the IM2.

Parameter	Value	Units
Supply voltage	220/460	V
Nominal speed (@60 Hz)	1140	rpm
Power	746	W
Nominal current (@460 V/60 Hz)	1.8	A

**Table 5:** Study cases for IM2.

Study Case	Label	Description
Healthy	HLT	Healthy induction motor
Unbalance	UNB	Unbalance in the motor rotor
Horizontal misalignment	HML	Motor misalignment and load on the same horizontal plane
Vertical misalignment	VML	Height gap between motor and load
Loose bolt	LB	Loose bolt in the bar that fixed the motor to the test bench

**Table 6:** FLIR Lepton 3.5 infrared sensor specifications.

Specification	Detail
Output matrix	160 × 120
Infrared range	8 µm to 15 µm
Emissivity	95%
Thermal sensitivity	50 mK

**Table 7:** Resource consumption per IPcore of thermal indicators.

Resource	Utilization (%)
LUTs	13.65
Registers	8.85
Slices	11.38
Logical LUT	12.63
Memory LUT	3.14
RAM block	3.57
DSPs	3.18

**Table 8:** Resource consumption per FFT IPcore.

Resource	Utilization (%)
LUTs	12.62
Registers	9.38
Slices	10.43
Logical LUT	8.86
Memory LUT	4.81
RAM block	6.07
DSPs	5.45

**Table 9:** Confusion matrix of the KNN classifier with thermal indicators from WEG motor.

		Predicted				
		HLT	MAMT	UNB	BDF	BRB
**True**	HLT	100%				
	MAMT		100%			
	UNB			97.8%		2.2%
	BDF				100%	
	BRB			0.6%		100%

**Table 10:** Confusion matrix of the SVM classifier with thermal indicators from WEG motor.

		Predicted				
		HLT	MAMT	UNB	BDF	BRB
**True**	HLT	100%				
	MAMT		100%			
	UNB			98.9%		1.1%
	BDF				100%	
	BRB			3.9%		96.1%

**Table 11:** Comparison chart of the proposed methodology against the state of the art in related literature for IM fault detection.

Reference	Proposed Indicators	Classification Method	Analyzed Faults	Acquired Signals	Accuracy Rate
Nayana and Geethenjali [5]	Statistical and non-statistical indicators.	Laplacian score (LS) and linear discriminant analysis (LDA)	Bearing faults	Vibration	98.94%
Toma and Kim [6]	Temporal statistical indicators	SVM, RF, and a KNN	Bering faults	Current	98%
Liang et al. [7]	Time domain and time-frequency features	Parallel convolutional neural networks	Bearing faults	Vibration	99.6%
Jiang et al. [8]	Particle swarm optimization, sample entropy, and time domain statistical features	Feature Incremental Broad Learning (FIBL)	Short-circuit, mechanical imbalance, bent rotor, bearing faults	Current and acoustic	92.73%
Shao et al. [11]	Time-frequency distribution	CNN	Rotor faults, bearing faults, shorted turns.	Vibration and current	99.8%
Cao et al. [12]	Pre-trained deep neural network	CNN and transfer learning	Gearbox-related faults	Vibration	99.41%
Jing et al. [13]	Frequency domain features	CNN	Gearbox-related faults	Vibration	98%
Camarena et al. [21]	Frequency domain features	Deep belief network	Stator short-circuit, unbalance, BRB, bearing faults	Vibration	99.9%
Ince et al. [22]	Time domain statistical indicators	GA, KNN, and decision tree	Bearing faults	Current	99%
Choudary et al. [15]	Two-dimensional discrete wavelet transform	SVM	Bearing faults	Infrared thermography	97.9%
Khanjani y Ezoji [16]	Automatic segmentation, CNN	KNN and SVM	Stator short-circuit	Infrared thermography	100%
Mahami et al. [17]	Bag-of-visual-word	Extremely randomized tree	Stator faults	Infrared thermography	100%
**Proposed method**	Mean and standard deviation thermal indicators	SVM and KNN	MAMT, UNB, Bearing-related, BRB	Infrared thermography	99.0%

## Data Availability

The data presented in this study are available on request from the corresponding author. The data are not publicly available due to other research works in progress using same data.
